# Energy Efficient Consensus Approach of Blockchain for IoT Networks with Edge Computing

**DOI:** 10.3390/s22103733

**Published:** 2022-05-13

**Authors:** Shivani Wadhwa, Shalli Rani, Kavita, Sahil Verma, Jana Shafi, Marcin Wozniak

**Affiliations:** 1 Chitkara University Institute of Engineering and Technology, Chitkara University, Punjab 140401, India; shivani.wadhwa@chitkara.edu.in; 2 Department of Computer Science and Engineering, Chandigarh University, Mohali 140413, India; kavita@ieee.org (K.); sahilverma@ieee.org (S.V.); 3 Department of Computer Science, College of Arts and Science, Prince Sattam bin Abdul Aziz University, Wadi Ad Dawasir 11991, Saudi Arabia; j.jana@psau.edu.sa; 4 Faculty of Applied Mathematics, Silesian University of Technology, 44-100 Gliwice, Poland

**Keywords:** blockchain, consensus, energy, edge, offloading

## Abstract

Blockchain technology is gaining a lot of attention in various fields, such as intellectual property, finance, smart agriculture, etc. The security features of blockchain have been widely used, integrated with artificial intelligence, Internet of Things (IoT), software defined networks (SDN), etc. The consensus mechanism of blockchain is its core and ultimately affects the performance of the blockchain. In the past few years, many consensus algorithms, such as proof of work (PoW), ripple, proof of stake (PoS), practical byzantine fault tolerance (PBFT), etc., have been designed to improve the performance of the blockchain. However, the high energy requirement, memory utilization, and processing time do not match with our actual desires. This paper proposes the consensus approach on the basis of PoW, where a single miner is selected for mining the task. The mining task is offloaded to the edge networking. The miner is selected on the basis of the digitization of the specifications of the respective machines. The proposed model makes the consensus approach more energy efficient, utilizes less memory, and less processing time. The improvement in energy consumption is approximately 21% and memory utilization is 24%. Efficiency in the block generation rate at the fixed time intervals of 20 min, 40 min, and 60 min was observed.

## 1. Introduction

Rapidly growing technologies based on Internet of Things (IoT) are gaining lot of attention because of advancements in sensor technologies and wireless communications. The impact of this development can be seen in various sectors, such as healthcare, various fields of businesses, industries, smart sector developments, etc. [1]. Security is a major concern in these fields as various heterogeneous devices are connected to each other.

Nowadays, a variety of IoT attacks threaten its acceptance. Most existing attacks are phishing attacks, malware, zero-day attacks, spyware, denial of service (DoS), etc. These attacks may be internal or external; active or passive. Each layer of the IoT suffers from at least one of these attacks. All layers of IoT suffers from DoS attacks, which prevent access to the network [2].

For providing trust, security, privacy, and confidentiality to the IoT networks, blockchain is considered indwelling security technology [3,4]. It offers solutions to solve many problems that exist in traditional distributed database systems. The hack-proof nature of blockchain provides security features to data being produced by IoT devices [5]. The data are encapsulated in the block; creation of the block takes place by the process known as mining. Mining of most widely used consensus approach, i.e., PoW, is an energy consuming process. One major reason for consuming an enormous amount of energy is the process of repeating cryptographic hashing until the hash of the fixed number of initial zeroes is generated [6]. The consumption of electricity for the purpose of mining of a PoW-based blockchain has become a hot topic for researchers. Hash calculation in the blockchain makes it energy-hungry. On average, the current hash rate is 205.322 terahashes per second [7]. Due to excessive hashes produced per second, tremendous energy is utilized for generating hashes.

Integrating blockchain with IoT networks ensures trusted data provision. Implementing blockchain approach provides the operation of services in a decentralized fashion, where there is no need for a third party to be involved as an intermediary [8]. Various non-colluding parties are not included in the blockchain network, which makes it best for providing trust to the IoT network.

The sensor nodes of IoT devices are generally low-powered due to which they need to offload the computation tasks to other devices. Mostly, offloading of computations is done with the adjacent edge devices, fog devices, cloud devices, multiparty service providers, etc. [9]. Edge computing provides computational capabilities by immediately lying very close to the end devices. The technologies that demand many computations can be provided with the computing resources by offering very low latency [10]. The main parameters to be considered while offloading are latency, energy consumption, network throughput, and resource allocation [11,12].

The application of blockchain-enabled edge-based IoT systems lies in e-healthcare [13], intelligent transportation systems [14], smart industries [15], etc. Much attention is given toward improving blockchain-based IoT systems by conducting modifications in the block size, the time interval between the block formation, improving consensus methods, resource utilization, etc. [16]. The energy consumption of mining processes of blockchains may impede its adoption in the IoT world.

Work was done to find the energy consumption of various consensus approaches, such as proof of work (PoW), stellar, and ripple for IoT architecture. The authors in [17] proposed a BlockAuth scheme to provide security, reliability, and fault tolerance in edge and IoT environments by designing consensus mechanisms and smart contracts. The model proposed by them is well applicable for the private or consortium blockchain platform but not for the public blockchain platform. The authors in [18] developed proof of deep learning (PoDL) for mobile edge computing networks. This work was done to replace the computation intensive PoW consensus approach. However, this approach is not yet implemented for delay or data sensitive cases, such as automatic driving. The authors in [19] proposed a proof of quality factor (PoQF) consensus algorithm for message passing in vehicular edge networks. However, the authors also claimed that delay could be further reduced by eliminating the voting based consensus approach. So, these exists the need for an efficient consensus mechanism, which can improve the performance evaluation parameters and make blockchain sustainable for IoT.

Our paper provides security to the IoT network by incorporating blockchain in its functionality. We modified the consensus approach of the blockchain by using resources from the edge network. Different specifications of the edge nodes are used to select the miner and the rest of the miners just perform the process of validation of the block. The novelty of our paper involves the selection of the single miner with good specifications. The main contribution of our work is summarized below:

1. Data generated by IoT devices is sent to the blockchain network for security purposes. IoT devices are incapable of performing extensive computations required for reaching consensus.
2. Highly capable devices of the edge network participate in reaching the consensus by becoming miners of the blockchain as computations from the blockchain environment are offloaded to the edge network.
3. Specifications of the devices on the basis of RAM, CPU, and the bandwidth of the network are used for the selection of the miner. Formula is also used for finalizing the miner amongst different capable miners.

This paper is organized as follows: Section 2 provides the overview of the related study. The system model is presented in Section 3 along with the components of the system, assumptions, and workflow of the system model. Section 4 presents the proposed model. Section 5 discusses the results of the experimental study. Section 6 concludes the work and provides insight into a future scope of the work.

## 2. Related Work

Satoshi Nakamoto in 2008 proposed blockchain technology by using the PoW consensus mechanism [6]. To obtain the significant performance using PoW consensus, there is a need to combine various mining pools. A lot of energy is consumed by these mining pools, which makes them impractical for low powered devices. They are mostly used for cryptocurrencies but now their applications can be seen in various fields of IoT, artificial intelligence, etc. Many researchers in the blockchain field are working to improve consensus for its easy applicability in various fields. This section provides a brief overview of the related work in the field of energy consumption, modifying the consensus approach of the blockchain.

### 2.1. Modifying the Consensus Approach of the Blockchain

In [20], a modified practical Byzantine fault tolerance approach was framed for a virtual power plant. This approach fastens the consensus mechanism by reducing the time and ensuring the fault tolerance. In [21], a consensus framework based on the Byzantine approach was proposed to enhance the security of data between the electric vehicles and distribution network. This approach also prevented the system from malicious attacks and vulnerable threats. For IoT networks, an improved statistical approach was provided in [22] to reach consensus on the basis of PoW. This technique is well applicable for cloud, fog, and edge networks. For IoT edge nodes, proof of authentication was designed so as to provide a solution to the resource-constrained devices of IoT [23]. A decentralized consensus approach was designed on the basis of voting for the consortium blockchain to reduce the energy consumption and time [24]. Assets and reputation are considered in this consensus approach for providing assets or penalizing the miners. The intelligent consensus approach was created for software-defined networks by using transfer learning [25]. The authors in [26] computed the trust score of all members of the cluster for the purpose of mining in blockchain. This consensus approach provided better throughput and energy.

### 2.2. Improving Energy Consumption Using Non-Consensus Approach

An improved energy-efficient technique is proposed for industrial IoT by jointly optimizing the device allocation and weighted cost. The problem is proposed as the Markov decision problem and solved by deep reinforcement learning. Mobile edge computing and blockchain are used to ensure the security to the system [27]. Fog consensus based on federated learning is achieved for vehicular networks [28]. This technique improves the performance parameters, such as accuracy, energy consumption, throughput, and latency. The authors in [29] proposed a cluster technique for IoT networks by using blockchain and SDN. This technique eliminates the need of the energy consuming consensus approach, which makes it better than existing blockchain strategies. Table 1 shows the related work on consensus approach. The framework based on the Lyapunov optimization is framed to maximize the revenue generated for edge services and minimize the energy consumption [30]. In [31], the authors proposed a deep reinforcement learning-based technique to finalize the offloading policy. Computations were offloaded to edge servers to provide services to the resource-constrained devices. The authors in [32] proposed an adaptive linear prediction technique to providing energy efficient techniques by charging coins in unmanned aerial vehicles. Table 2 summarizes the literature survey on energy efficient techniques.

From the literature survey performed, mostly on the IoT environment, it is evident that the blockchain network can provide security to the IoT data. However, blockchain further needs assistance from edge, fog, or cloud computing to perform the complex computations of the consensus approach. Thus, it is essential for the blockchain network to offload its computation to other networks in case of resource-constrained IoT networks. Our approach also designs an efficient consensus approach by using edge networks and minimizes the energy consumption of the miners. The specifications of the edge devices, such as random access memory (RAM), central processing unit (CPU), and bandwidth, are used for selecting miners, which improves the performance of our model.

## 3. System Model

This section discusses the components, assumptions, and flowchart of the proposed system.

### 3.1. Components of the System

The IoT devices, blockchain network, transaction pool, smart contract, edge cluster, and edge nodes are the main components of the system.

- IoT devices: the IoT devices mainly consist of sensors, actuators, radio frequency identification system (RFID), etc., for the collection of environmental data. The sensor nodes are used for sensing the data according to their specialization. The IoT devices in the proposed model may consist of devices of smart city, smart agriculture, smart industry, etc.
- Blockchain network: the blockchain network stores the IoT data securely in a distributed and decentralized manner.
- Transaction pool: the data generated by IoT devices are stored in a transaction pool. The miners collect data from the pool for the creation of block.
- Smart contract: the smart contract contains the information of the authenticity of nodes. Smart contracts are referred every time before finalizing the edge cluster head.
- Edge cluster: edge clusters are formed at edge networks. Each edge cluster consists of a cluster head, which is formed randomly on the basis of the formula mentioned in the next section.
- Edge nodes: edge nodes are the part of the edge network. All edge nodes are capable of performing complex computational tasks of reaching consensus in the blockchain.

### 3.2. Assumptions

The assumptions of our system model are:

- All edge nodes possess high computational power.
- All edge nodes and blockchain nodes are authentic.
- Smart contract stores the information of authenticity of nodes.
- All nodes of the blockchain network possess high storage capacity.

### 3.3. Workflow of the System Model

**Figure 1:**
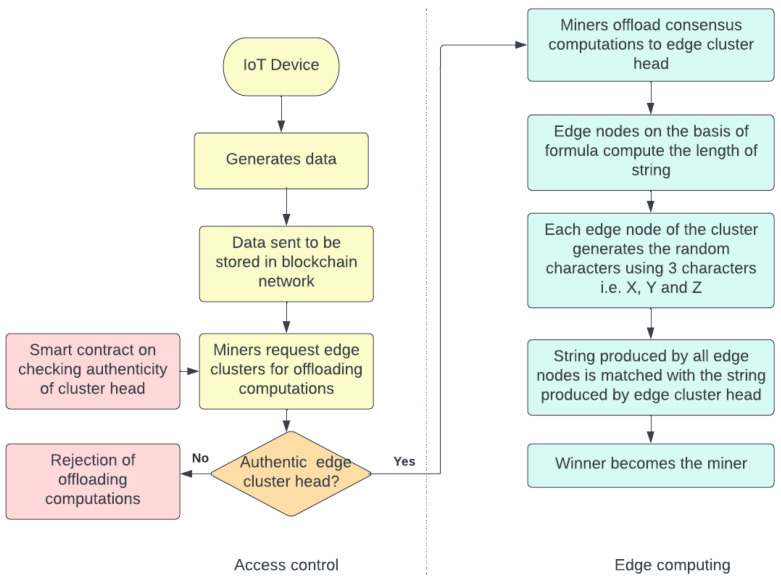
Workflow of the system model.

Figure 1 describes the workflow of the system model. Two sections of the system model are created, i.e., access control and edge computing. The access control section performs the task of verification of edge nodes where the task of mining is transferred. This verification is done by checking the authenticity, where information is stored in smart contracts. The edge computing section consists of the method of selecting the appropriate miner for the purpose of mining.The selection of the miner is done on the basis of matching the strings of all edge nodes with the edge cluster head. The edge node with the maximum number of matches becomes the miner and starts computing the nonce field of the block for obtaining the desired block hash. The block is then sent to other candidate miners for the process of validation. After validation, incentives are given to the miner by the edge cluster head for the successful creation of a block. This completes the final consensus in the blockchain network.

## 4. Proposed Method

Adverting toward the tremendous energy consumption and delay in the processing time of the consensus mechanism, to obtain the block approved for its inclusion in the blockchain, it is important to improve the existing consensus approach used in the blockchain environment. As IoT data approach the blockchain network for its security, it is the responsibility of the blockchain miners to collect all the events in a pool and then store them in the block. The blockchain miners use the resources of the edge networks for the purpose of mining.

The edge networks contain edge nodes with good mining capacity. However, in our approach, all edge nodes were not used for mining. One of the edge node was selected amongst all the candidates of edge nodes, which could act as a miner. The specifications, such as random access memory (RAM), central processing unit (CPU), and bandwidth of the candidate miners, are basically used for selecting the actual miner of the block. Devices with good specifications mean that they are highly capable of performing the computations. This is the reason for selecting the specifications of the edge device for reaching consensus of the blockchain.

**Figure 2:**
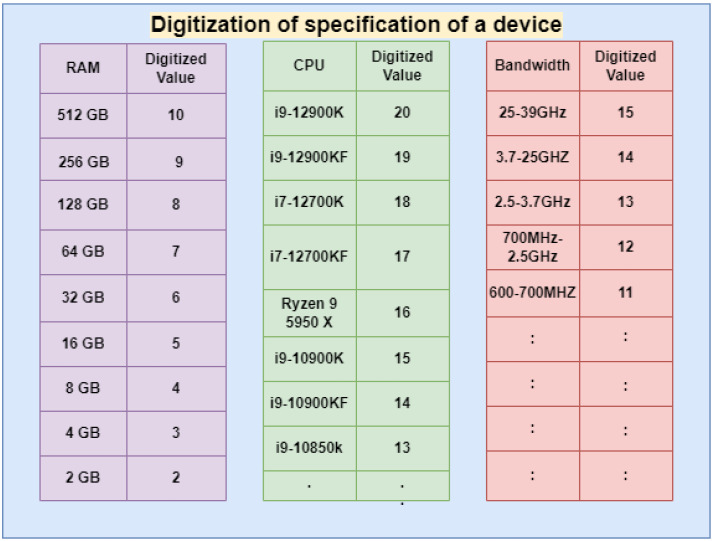
Random sample, digitization of specifications of various devices.

**Figure 3:**
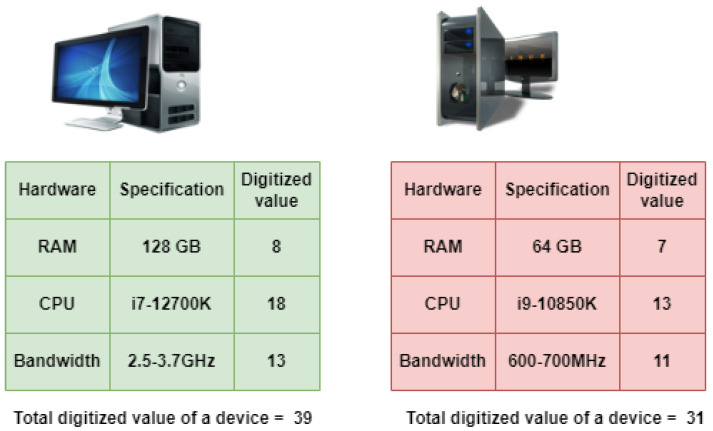
Example of digitization of specifications of a device.

**Figure 4:**
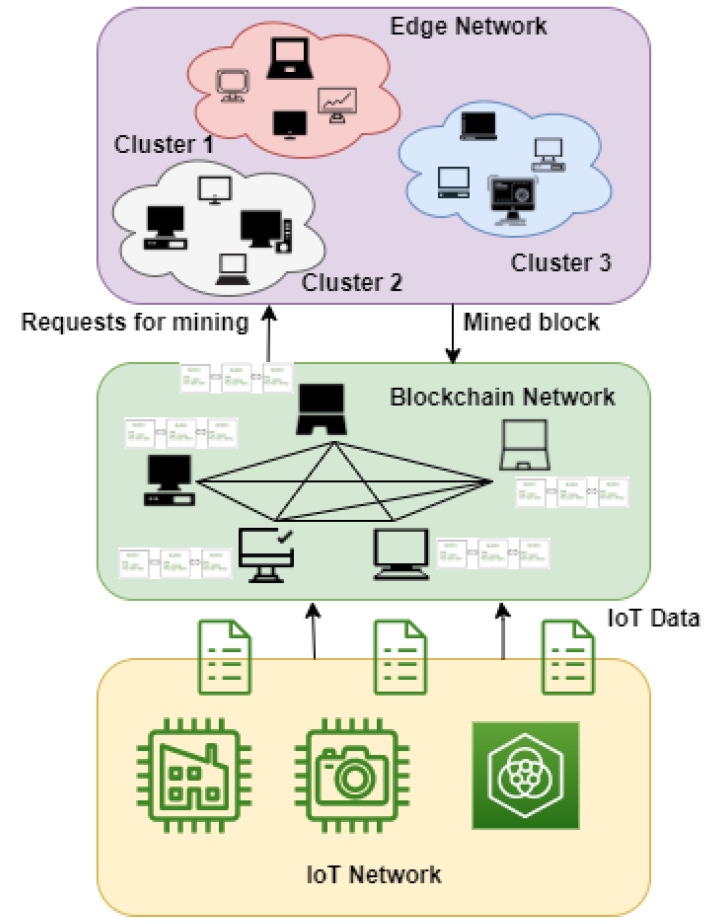
Proposed model.

Our proposed consensus approach can minimize the energy consumption of the devices by selecting one miner instead of all miners solving the same mathematical puzzle. This approach also reduces the maintenance costs of the edge devices. The specifications of the devices are digitized by assigning numbers to the devices on the basis of random access memory (RAM), central processing unit (CPU), and bandwidth. The specifications of various hardware are assigned some numbers to digitize the system. The specifications with high resources are provided with the greatest number. The process of digitizing the different hardware types is shown in Figure 2. This process of digitizing the devices is done periodically after 12 hrs, to update the number assigned to them and give a chance to other devices to become miners. The example of digitization of two devices that can act as miners at edge networks is shown in Figure 3. The sums of the digitized values of given devices are 39 and 31. Each cluster node contributes 7% of the sum to the edge cluster head. The purpose of this contribution is to provide incentives to the miner on the basis of collection being done. The proposed method can be observed from Figure 4. This contribution also prevents malicious nodes from taking part in mining, as they need to contribute. This sum is used to find the length of the string to be generated by the devices. The formula for computing the length of the string, *l* is:

(1) $$l=(\left(sum^{2}\right)\;\mathrm{mod}\;n)+3$$

where,

*n* is the number of miners in the edge cluster;
*s* is the sum of the digitized values of a given device.

On the basis of this length, each node present in the cluster uses the random function to find the random string by using three characters, i.e., X, Y, and Z. The node with the maximum value of the sum and contribution given becomes the suitable candidate for becoming the cluster head. Authenticity of the edge cluster head is verified through the smart contract. The smart contract eliminate the chance of malicious nodes becoming the edge cluster head. All edge clusters behave in the same manner and the one that responds quickly receives the approval of the mining block. The edge cluster head also computes the random string by using three characters, i.e., X, Y, and Z. All edge nodes compare the random string with the random string of the edge cluster head. The edge node with the maximum number of corresponding mappings becomes the miner. Two string matching approaches have been used for comparing the strings, i.e., the brute force approach and Boyer–Moore approach. Through our proposed approach, only the miner will perform the task of solving the complex mathematical puzzle, as solved in PoW. Our approach performs better as all nodes are not doing the computation tasks. In this way, the computational tasks of various miners are reduced.

## 5. Results and Discussions

In this section, the performance of our proposed model, using brute force and Boyer–Moore, is evaluated and compared with other consensus approaches i.e., PoW and PoS.

### 5.1. Experimental Setup

Python language was used to simulate the behavior of the blockchain platform. The Ethereum platform was used for the implementation of the blockchain environment. The blockchain parameters were: block size was 1 MB, the elliptic curve digital signature algorithm was used for cryptographically linking the blockchain, PoW and PoS consensus approaches were considered for the comparison. The experiment was conducted on Core i7-8565U CPU 1.80 GHz, 1992 Mhz, 4 Core(s), and 8 logical processor(s). The edge environment was used for offloading computations. Devices on edge are dynamic in nature, i.e., they are of different specifications. The multi-threading procedure was used to create multiple nodes of the blockchain. In our experiment, the number of blocks generated by different consensus approaches in 20, 40, and 60 min, memory utilization, and energy consumption, were taken into consideration.

### 5.2. Efficiency of Block Generation

**Figure 5:**
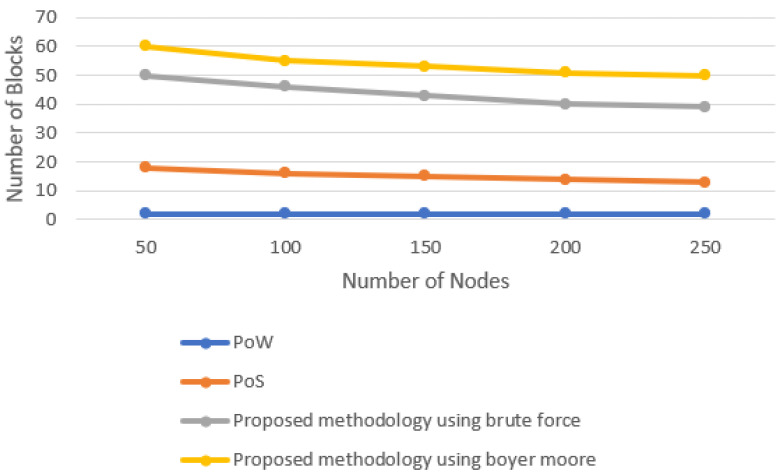
Block generation for 20-min time interval.

**Figure 6:**
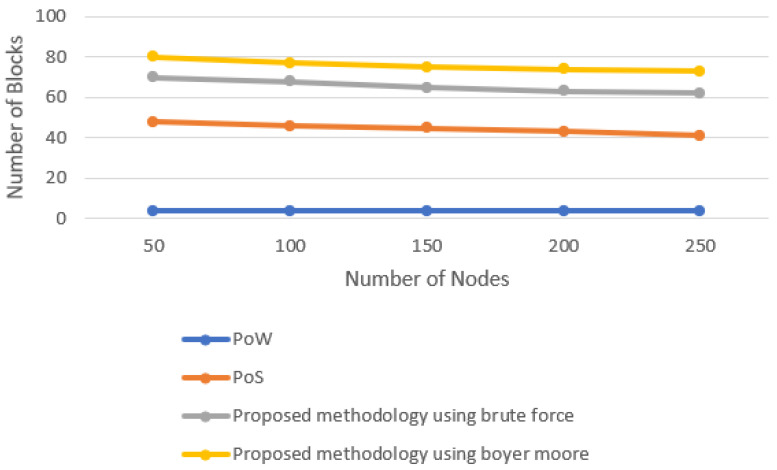
Block generation for 40-min time interval.

**Figure 7:**
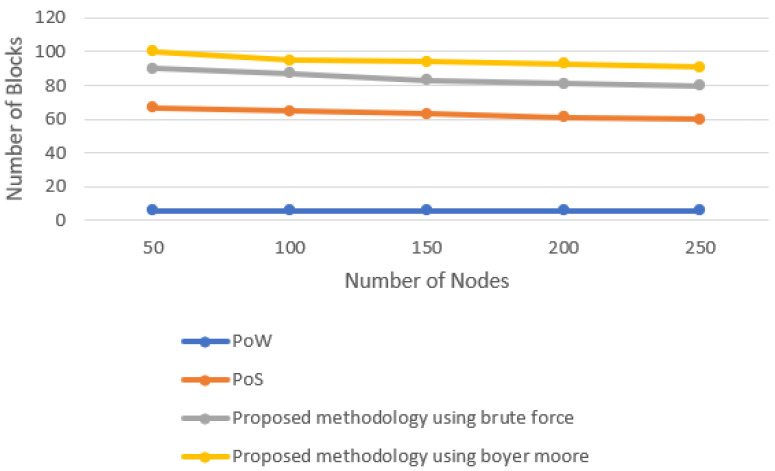
Block generation for 60-min time interval.

The number of blocks produced by different consensus algorithms and the proposed consensus approach is evaluated in this section. The number of nodes are set to 50, 100, 150, 200, and 250 by using the concept of multi-threading. Figure 5 shows the line graph when the time limit is set to 20 min. The number of blocks increases with the increase in time. However, it was observed that, in the fixed time limit, the number of blocks produced by varying the number of nodes almost remained constant as it was the task of a single miner to produce the block. Figure 6 shows the line graph when the time limit was set to 40 min. With the increase in the time limit, the number of blocks produced also increased. Figure 7 shows the line graph when the time limit was set to 60 min. By comparing these data in all three scenarios, it is observed that the proposed model performs better. The proposed model, in the case of Boyer–Moore, performs better because it is less complex in performing string comparisons.

### 5.3. Memory Utilization

**Figure 8:**
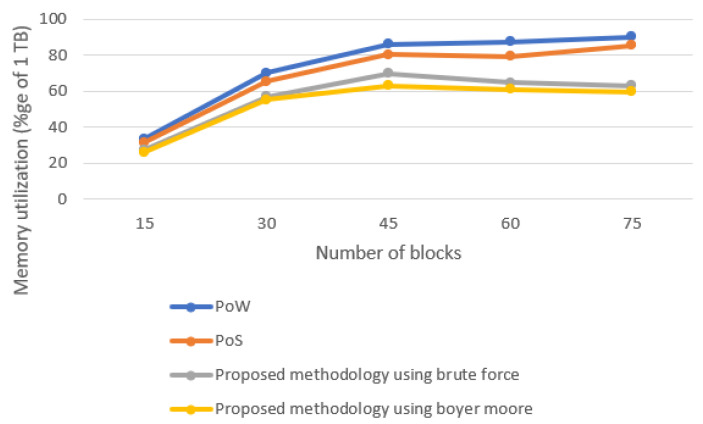
Memory utilization of different consensus approaches.

This section discusses the memory utilization of the different consensus algorithms. Figure 8 shows the trend of memory utilization by different consensus approaches. The experiment was conducted on 1 TB of memory. Memory utilization of all consensus algorithms increases with the increase in the number of blocks. Our proposed technique almost shows equivalent memory utilization because of the slight difference in the string matching technique. Memory consumed by a single miner in our proposed technique remains the same. The memory consumed by PoW is highest because all miners start solving the complex puzzle.

### 5.4. Energy Consumption

**Figure 9:**
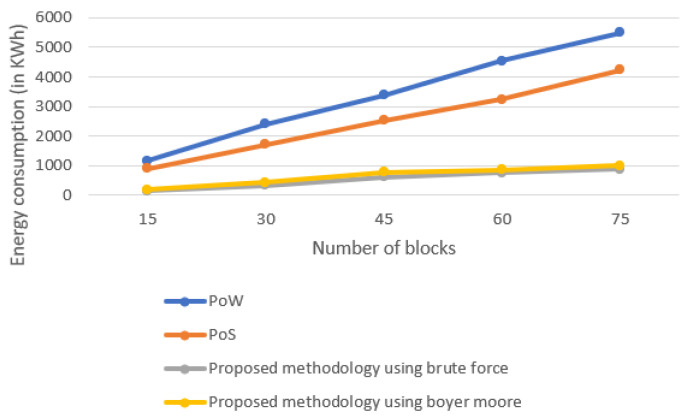
Energy consumption of different consensus approaches.

This section provides insight into the energy consumption by different consensus approaches. Figure 9 clearly shows the change in behavior of the energy consumption by different consensus approaches. It is clear from the figure that energy consumption of PoW is the highest, as various miners together solve the complex mathematical puzzle. PoS comparatively consumes less energy because of the selection of the miner and the process to select the miner. Our proposed technique consumes much less energy because of the selection of the single miner for the mining of the blockchain. Almost equivalent energy is consumed in case of brute force and Boyer–Moore.

## 6. Conclusions

Security and efficient utilization of energy are main challenges of the IoT network because of its resource-constrained devices. Blockchain provides security to the IoT network but its consensus mechanism is an energy-hungry mechanism. Due to high energy consumption for the process of consensus, blockchain miners offload their processing tasks to the edge network. At the edge networks, edge clusters are formed, where there is a collection of miners with good specifications for performing mining. Within edge clusters, ’game’ is played to finalize the miner. The miner is selected on the basis of the digitization of the specifications of the respective machines. In game, brute force and Boyer–Moore string processing algorithms are used. The selected miner performs the mining and eliminates all other miners to perform the task of mining. The experiment is conducted in Python on the Ethereum platform for the purpose of evaluation. The proposed model makes the consensus approach more energy efficient, utilizes less memory, and less processing time. There was improved energy consumption, by approximately 21%, and memory utilization by 24%. Efficiency in the block generation rate at fixed time intervals of 20, 40, and 60 min was evaluated; it was observed that the proposed model performed better than other consensus approaches. The limitation of this work can be visualized when (near) similar types of hardware are present in the edge network. This work can be extended in the future by implementing this experiment in real world entities. This work can also be implemented on the consortium blockchain, to enhance this approach for multiple blockchain environments.

## Figures and Tables

**Table 1:** Related works on the modification of consensus approach.

Reference No.	Consensus Approach Used	Contributions	Validation Parameters	Future Scope
[20]	Practical Byzantine fault tolerance	Proposed blockchain network collaboration mechanism	Time and fault tolerance	Use of multichain and sidechain to improve the performance of model
[21]	Framework based on the Byzantine approach	Energy trading process is formulated by using the Byzantine general approach	Success probability of attack	Refining the consensus approach
[22]	Modified proof of work	Proposed novel algorithm for reaching consensus by using polynomial matrix factorization and statistical likelihood maximization	memory usage, energy, convergence time, and energy consumption	Using smart contract for its adaptability
[23]	Proof-of-authentication	Consensus designed for resource-constrained IoT devices	Energy and latency	Consideration of transparency and security of IoT architecture
[24]	Proof of reputation, proof of assets	Decentralized consensus approach is designed on the basis of voting	Time and energy	Suitable for complex scenarios
[25]	Application aware consensus	Virtualized consensus approach using transfer learning	Throughput, energy, and time	Adapting edge artificial intelligence for blockchain
[26]	Circle of trust–consensus	Use of trust scores	Throughput and energy	

**Table 2:** Related work on improving energy efficiency.

Reference No.	Technique Used	Contributions	Validation Parameters	Future Scope
[27]	Practical Byzantine fault tolerance	Energy-efficient technique for industrial IoT by jointly optimizing the device allocation and weighted cost	Energy consumption, total time, and computation overhead	Considering other consensus approaches
[28]	Consensus based on federated learning (FL)	Achieved fog consensus using FL for vehicular networks	Accuracy, energy consumption, throughput, and latency	Adopting different FL techniques
[29]	Use of SDN controllers	Cluster techniques for IoT networks by using blockchain and SDN	Energy, throughput, and time	High-level blockchain architecture
[30]	Offloading computations to mobile edge computing servers	Framework based on the Lyapunov optimization is framed	Response time and energy consumption	Implementation on real-world networks based on blockchain
[31]	Offloading computations to mobile edge computing servers	Deep reinforcement learning technique is used to finalize the offloading policy	Processing delay and energy consumption	Considering offloading requirements of various IoT devices due to the increase in network traffic
[32]	Adaptive linear prediction technique	Charging coins are obtained by unmanned aerial vehicles	Accuracy and energy consumption	

## Data Availability

Not applicable.
